# Tumor Budding as a Risk Factor for Lymph Node Metastasis and Local Recurrence in pT1 Colorectal Cancer: A Systematic Review and Meta-Analysis

**DOI:** 10.1016/j.gastha.2025.100713

**Published:** 2025-05-27

**Authors:** Heng Zhang, Femke Simmer, Alessandro Lugli, Iris D. Nagtegaal

**Affiliations:** 1 Department of Pathology, Radboud University Medical Center, Nijmegen, Gelderland, The Netherlands; 2 Institute for Tissue Medicine and Pathology, University of Bern, Bern, Switzerland

**Keywords:** pT1 Colorectal Cancer, Tumor Budding, Lymph Node Metastasis, Local Recurrence

## Abstract

**Background and Aims:**

Implementation of population screening programs resulted in an increase in early colorectal cancer (CRC, pT1). For these small CRC, endoscopic local resection is the preferred treatment. However, the presence of lymph node metastasis (LNM) and risk of local recurrence might require additional surgery. Tumor budding (TB) is a well-known biomarker for adverse outcomes in pT1 CRC. This study aims to further explore the relationship between TB and in pT1 CRC and to clarify the relationship between TB and local recurrence, to provide more strategies for the selection of surgical treatment.

**Methods:**

A systematic review was conducted using the MEDLINE and EMBASE databases to investigate the relationship between TB and LNM and local recurrence in pT1 CRC. Meta-analysis based on a random-effects model was performed to evaluate the relationship between TB and these 2 factors.

**Results:**

A total of 57 observational studies were included in the meta-analysis, with a total of 24,956 patients. High-grade TB was significantly associated with both LNM (risk ratio (RR) = 4.04, 95% confidence interval (CI), 3.52–4.64, I^2^ = 56.11%, *P* < .001) and local recurrence (RR = 2.35, 95% CI, 1.21–4.54, I^2^ = 26.18%, *P* = .01). Sensitivity analysis confirmed the robustness of our pooled results. Subgroup analysis also explored possible sources of heterogeneity. For LNM, geographical location (Asia: RR = 4.22, 95% CI, 3.64–4.89; Others: RR = 2.72, 95% CI, 2.08–3.57, *P* = .01) and year of publication (<2015: RR = 4.96, 95% CI, 4.01–6.15; ≥2015: RR = 3.58, 95% CI, 2.99–4.29, *P* = .02) showed significant differences in the subgroup analysis. We cannot rule out publication bias for LNM risk.

**Conclusion:**

Our findings confirm that TB is a strong predictor of local recurrence, but in particular of LNM in pT1 CRC and offers effective guidance for selecting further surgical treatment strategies.

## Graphical abstract

**Figure:**
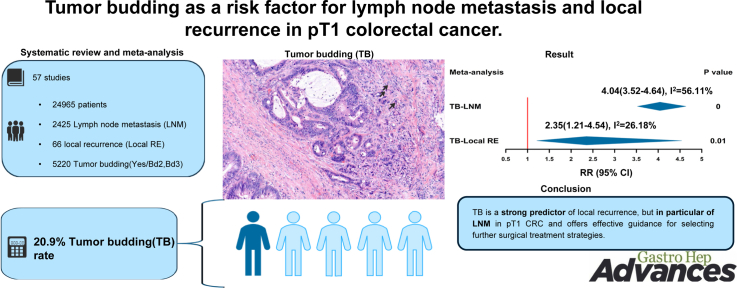

## Introduction

Colorectal cancer (CRC) is the third most common cancer and the second leading cause of cancer-related death worldwide.^1^ With the introduction of population screening programs, the incidence of early CRC is increasing year by year, currently accounting for 40% of CRC detected by screening.^2^, ^3^, ^4^

Early CRC confined to the submucosa (pT1) is preferably treated by endoscopic resection,^5^, ^6^, ^7^ since it has lower morbidity, mortality, and cost than surgery. However, lymph node metastases (LNMs) occur in about 10%–15% of pT1 patients,^8^^,^^9^ and local recurrence in approximately 2%.^10^ For these high-risk patients, additional surgical treatment can be considered.^10^^,^^11^ To identify the high-risk patients, several risk factors have been proposed including invasion depth, differentiation grade, specific subtypes (signet ring cell carcinoma, or mucinous carcinoma), small vessel invasion, and tumor budding (TB).^12^ TB, defined as a single cell or a cluster of up to 4 cells at the invasive margin of CRC^13^ is a marker for aggressive tumor biology and closely related to LNM, recurrence, and poor prognosis in CRC.^14^, ^15^, ^16^, ^17^

Despite previous variation in methodology for TB assessment, previous meta-analyses and systematic reviews have identified TB as one of the relevant biomarkers in pT1 CRC.^18^, ^19^, ^20^ As a consequence, TB is now incorporated into reporting guidelines for pT1 CRC.^21^^,^^22^ In 2016 the international tumor budding consensus conference standardized assessment methods for TB.^13^ Now, several years after the consensus meeting, we aim to further clarify the relationship between TB and LNM in pT1 CRC with a focused meta-analysis. This focused approach allows us to explore the specific features of TB, including methodological aspects, based on the consensus definition, in contrast to both general meta-analyses of LNM risk in pT1^19^ and earlier focused reviews.^20^ In this study, we aim to expand the sample size to further validate the relationship between TB and LNM and to investigate the potential association between TB and local recurrence.

## Methods and Materials

This meta-analysis is performed according to the preferred reporting items of the Guidelines for Systematic Reviews and Meta-Analysis.^23^ This meta-analysis study protocol has been registered in the International Prospective Systematic Review Registry (Protocols.io https://dx.doi.org/10.17504/protocols.io.81wgbx6rolpk/v1).

### Search Strategy

The literature search was carried out in consultation with the Radboudumc librarian, and the corresponding searches of the electronic literature databases MEDLINE and EMBASE were from inception to May 24, 2023. Supplementary material (Table A1) describes the search strategy and procedure in detail. We also manually cross-referenced relevant papers.

### Inclusion and Exclusion Criteria

In this meta-analysis, eligible studies needed to meet the following criteria: (1) pT1 CRC (2) Evaluate the relationship between TB and LNM and/or local recurrence. (3) Obtain diagnosis of TB by histopathology. (4) Provide the corresponding risk ratio (RR) or provide sufficient patient information to calculate RR value. (5) For studies with overlapping study cohorts, the study with the largest number of patients was chosen. The exclusion criteria that research needed to meet were as follows: (1) In vitro or animal studies, letters, meta-analysis, reviews, case reports, conference abstracts, Editorial, Erratum. (2) Non-English articles. (3) No full text available. (4) Studies with less than 50 eligible cases. (5) Studies with the same cohort source.

### Data Extraction

Two independent reviewers (H.Z. and F.S.) assessed the titles and abstracts of all literature retrieved. Irrelevant and duplicate studies were excluded, and for the remaining studies, the full text was screened using the inclusion and exclusion criteria. Any disagreements were resolved through consultation and discussion. From the final selected studies, the number of patients per group (TB + LNM+; TB + LNM-; TB-LNM+ and TB-LNM-) was collected to calculate the RR for LNM, and likewise for the RR for local recurrence. For studies that provided only the Odds ratio (OR) and partial patient information, we derived the missing patient data using the OR calculation method and then calculated the RR with the RR calculation method.^24^ Additionally, the following features, when available, were extracted: first author, year of publication, study country, period of diagnosis, total number of patients, gender statistics, mean age of the cohort, cancer site statistics, TB assessment method including staining, magnification, magnified area and the cut-off value, the total number of LNMs, total number of TB, and total number of relapsed patients.

### Quality Assessment

The quality of the literature was assessed using a checklist for standardization of histopathology studies.^25^ For assessment of studies with lymph node metastases, 13 out of the 20 proposed items were scored, for local recurrence 15 out of 20 (Table A2). The quality scoring standards for each study can be divided into 1, 0.5, or 0, and each score corresponds to good, poor, and not, respectively. Studies with a score ≤ 60% were considered low-quality documents, 60%–80% are medium-quality documents, and more than 80% are high-quality documents.

### Statistical Analysis

All statistical analyses were performed on dichotomous data using Stata software (version 17.0, Stata Corporation, College Station, Texas, USA). The association between TB and LNM and local recurrence was evaluated by RR and 95% confidence interval (CI). The Der-Simonian and Laird random-effects model was used for meta-analysis. *P* < .05 was considered statistically significant. Between-study heterogeneity was assessed using I^2^.^26^ A percentage higher than 50% was considered to indicate heterogeneity. To explore the stability of the meta-analysis, we conducted a sensitivity analysis by sequentially excluding individual studies (leave-one-out method) from the pooled results. Additionally, we performed sensitivity analyses by excluding one specific category of studies at a time. Moreover, subgroup analyses were performed to explore the sources of heterogeneity and to assess the impact of study variables on the results. Publication bias was assessed by visual inspection of funnel plots, with asymmetrically sloped funnels indicating publication bias and symmetrical ones indicating no compliance bias. The Egger test was performed, with *P* < .05 suggesting potential publication bias. Additionally, the trim and fill method was used to explore the impact of potentially missing studies on the overall effect-size estimate.

## Results

**Figure 1:**
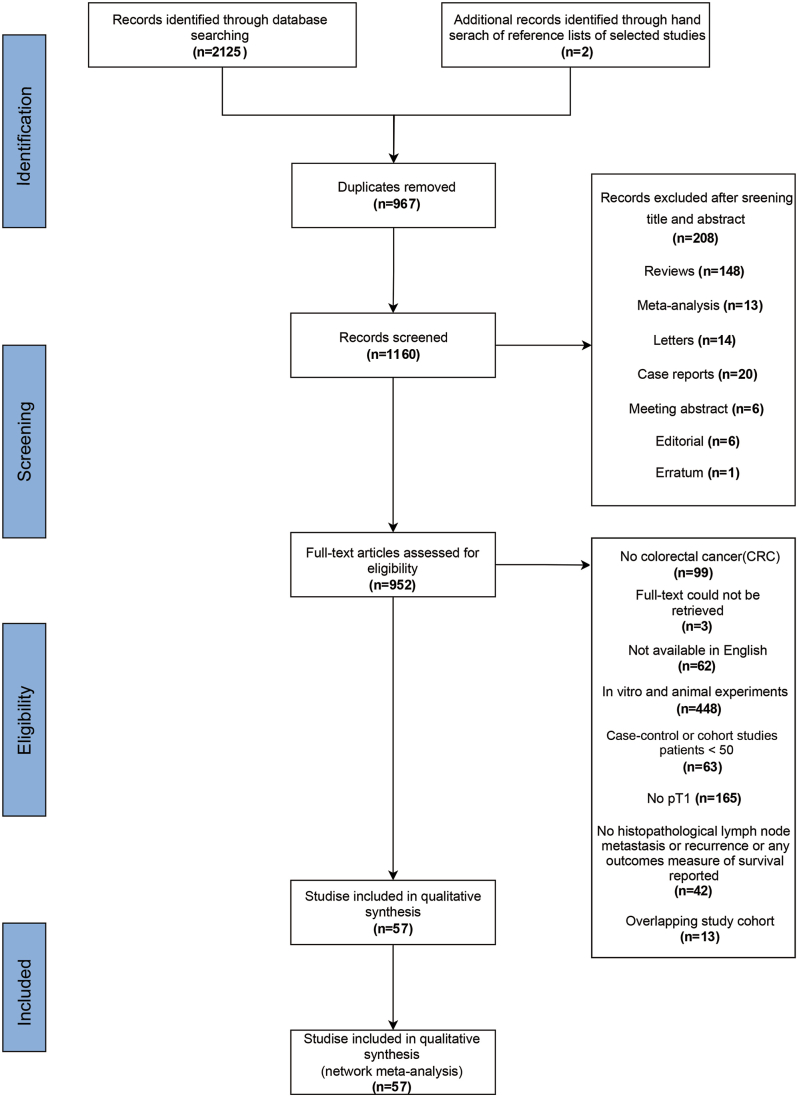
Flow chart of selection process.

We retrieved a total of 2125 papers through electronic databases and identified another 2 through manual cross-referencing. After excluding duplicates, there were 1160 remaining papers. Another 208 papers were excluded after title and abstract screening, and 895 studies were excluded after full-text analysis (for details see Figure 1). Ultimately, 57 studies met our inclusion criteria. Among them, 53 studies explored the relationship between TB and LNM,^11^^,^^27^, ^11^, ^27^, ^28^, ^29^, ^30^, ^31^, ^32^, ^33^, ^34^, ^35^, ^36^, ^37^, ^38^, ^39^, ^40^, ^41^, ^42^, ^43^, ^44^, ^45^, ^46^, ^47^, ^48^, ^49^, ^50^, ^51^, ^52^, ^53^, ^54^, ^55^, ^56^, ^57^, ^58^, ^59^, ^60^, ^61^, ^62^, ^63^, ^64^, ^65^, ^66^, ^67^, ^68^, ^69^, ^70^, ^71^, ^72^, ^73^, ^74^, ^75^, ^76^, ^77^, ^78^ seven studies explored the relationship between TB and recurrence^10^^,^^11^^,^^43^^,^^64^^,^^79^, ^80^, ^81^ and 3 studies simultaneously explored the relationship between TB and LNM and local recurrence.^11^^,^^43^^,^^64^

### Background

The characteristics of all the included studies are summarized in Table 1. All 57 studies adopted a retrospective observational design, and a total of 24,956 patients with pT1 CRC were included. Study groups ranged from 53 to 3556 patients. Three studies included rectal cancer only^43^^,^^65^^,^^81^ and 54 studies included colorectal cancer (in 10 studies, colon and rectal cancer could not be separately analyzed). Among them, 47 studies were conducted in Asia (23,051 patients, 92.4%), 8 studies were conducted in Europe (1710 patients, 6.9%)^11^^,^^28^^,^^40^^,^^43^^,^^60^^,^^63^^,^^81^ and 2 studies were conducted in North America (195 patients, 0.7%).^30^^,^^46^

**Table 1:** Characteristics of the Studies Included in the Meta-Analysis

Author	Year	Area	Period	n	High grade	Histological staining	Magnification	Area	Male (%)	Age(mean)	Location (rectum)%
Araki	1993	Japan	1980–1992	87	Yes/No	HE	400×	NA	NA	NA	59.8%
Hase	1995	America	1970–1985	79	Yes/No	HE	50×	NA	67.1%	60.7	27.8%
Tsuruta	2000	Japan	1995–1999	77	Yes/No	HE	NA	NA	72.7%	63	20.8%
Shimomura	2004	Japan	1978–1999	171	Yes/No	HE	100×	NA	63.7%	61.2	43.9%
Wang	2005	China	1969–2002	159	Yes/No	HE	NA	NA	67.3%	64.9	47.2%
Kazama	2006	Japan	1990–2001	56	Yes/No	IHC	50×	NA	73.2%	62.8	25%
Yasuda	2007	Japan	NA	86	Yes/No	HE	NA	NA	66.3%	67.03	20.9%
Yamauchi	2008	Japan	1991–2001	164	Bd2/Bd3	HE	200×	NA	62.8%	62.4	29%
Choi	2008	Korea	1989–2004	168	Yes/No	HE	200×	NA	58.9%	57	57.1%
Suzuki	2009	Japan	1990–2004	124	Bd3	HE	200×	NA	68.6%	63.6	34.7%
Ogawa	2009	Japan	1995–2003	83	Bd3	IHC	200×	NA	61.2%	62.5	NA
Komori	2010	Japan	1990–2004	111	Bd2/Bd3	HE	200×	NA	53.2%	61.2	36.9%
Tateishi	2010	Japan	1992–2005	322	Yes/No	HE	NA	NA	67.1%	61.4	56.2%
Sung	2010	Korea	1998–2009	76	Bd3	NA	200×	NA	47.4%	61.1	40.8%
Akishima-Fukasawa	2011	Japan	1989–2009	111	Bd2/Bd3	HE	200×	NA	68.5%	67.72	45%
Wada	2012	Japan	1995–2005	120	Bd2/Bd3	HE	20× (objective lens)	NA	68.3%	64.6	43.3%
Nakadoi	2012	Japan	1981–2008	499	Bd2/Bd3	HE	200×	NA	NA	63	23.6%
Doornebosch	2012	Netherlands	1996–2008	62	Bd2/Bd3	NA	20× (objective lens)	NA	56.5%	69	100%
Suh	2013	Korea	2007–2012	75	Bd3	HE	200×	NA	NA	NA	NA
Umemura	2013	Japan	1984–1999	142	Bd2/Bd3	IHC	200×	NA	66.2%	65	35.2%
Oka	2013	Japan	1981–2008	118	Bd2/Bd3	HE	200×	NA	NA	60.8	100%
Ryu	2014	Korea	2003–2012	179	Bd2/Bd3	HE	200×	0.785 mm^2^	64.2%	63.4	35.8%
Ueno	2014	Japan	1980–2011	3556	Bd2/Bd3	HE	20× (objective lens)	NA	61.1%	64.7	NA
Barresi	2014	Italy	NA	101	Bd2/Bd3	HE	200×	NA	58.4%	69.5	22.8%
Nishida	2014	Japan	2000–2011	265	Bd2/Bd3	HE	200×	NA	60.8%	65.1	44%
Yoshii	2014	Japan	1989–2008	389	Bd2/Bd3	NA	200×	NA	61.4%	63.9	14.1%
Macias-Garcia	2015	Spain	2000–2011	97	Bd3	NA	250×	NA	62.9%	68	30.9%
Miyachi	2015	Japan	2001–2014	653	Bd2/Bd3	HE	20× (objective lens)	NA	62.5%	65.1	29%
Kawachi	2015	Japan	1976–2007	806	Bd2/Bd3	HE	200×	0.95 mm^2^	59.8%	63.9	35.2%
Li	2016	China	2007–2011	57	Bd3	HE	NA	NA	67.7%	65.7	36.8%
Okamura	2016	Japan	1981–2009	265	Bd2/Bd3	IHC	20× (objective lens)	NA	59.6%	68	29.8%
Debove	2016	France	2000–2014	67	Bd3	IHC	40× (objective lens)	NA	NA	NA	100%
Pai	2017	America	2010–2014	116	Bd2/Bd3	HE	200×	0.95 mm^2^	53.5%	63.8	NA
Yim	2017	Korea	2000–2015	252	Bd2/Bd3	HE	20× (objective lens)/400×	NA	51.8%	61.8	39.3%
Tamaru	2017	Japan	1992–2008	701	Bd2/Bd3	NA	200×	NA	59.2%	66.2	25%
Lee	2018	Korea	2010–2016	133	Bd2/Bd3	HE	200×	NA	62.4%	63.7	36.1%
Makimoto	2019	Japan	2010–2018	53	Bd2/Bd3	HE	NA	NA	45.3%	68.2	37.7%
Takamatsu	2019	Japan	2005–2010	318	Bd3	IHC	20× (objective lens)	0.785 mm^2^	57.2%	63.62	NA
Ryul Oh	2019	Korea	2001–2016	1555	Bd3	NA	200×	NA	62.2%	64.8	34.6%
Yasue	2019	Japan	2005–2016	846	Bd2/Bd3	HE	20× (objective lens)	NA	55.6%	66	28%
Zhang	2019	China	2008–2014	290	Bd2/Bd3	HE	200×	0.785 mm^2^	54.3%	59.7	66.9%
Barel	2019	France	2009–2013	309	Bd2/Bd3	HE	NA	0.785 mm^2^	59.9%	68	29.5%
Mochizuki	2020	Japan	2001–2018	745	Bd2/Bd3	HE	20× (objective lens)	NA	61.7%	NA	NA
Cappellesso	2020	Italy	2009–2015	102	Bd2/Bd3	HE	400×	NA	59.8%	62.2	NA
Oishi	2020	Japan	1998–2015	217	Bd2/Bd3	HE	NA	NA	67.3%	66.9	NA
Yan	2021	China	2013–2020	141	Bd3	IHC	NA	NA	54.6%	62.3	40.4%
Kang	2021	Korea	2004–2011	221	Bd2/Bd3	HE	200×	NA	55.7%	NA	41.6%
Jin	2021	China	2006–2021	1194	Yes/No	NA	NA	NA	78.3%	49	52.8%
Kim	2022	Korea	2002–2019	395	Bd2/Bd3	HE	200×	NA	64.6%	63.5	33.2%
Aizawa	2022	Japan	2002–2012	339	Bd2/Bd3	HE	NA	NA	63.3%	68	NA
Gambella	2022	Italy	2010–2019	207	Bd2/Bd3	HE	200×	0.785 mm^2^	44.9%	70	19.3%
Tsuchihashi	2022	Japan	1984–2012	526	Yes/No	NA	NA	NA	NA	NA	37.1%
Ha	2022	Korea	2001–2014	547	Bd3	NA	200×	NA	62.9%	60	36.2%
Ozeki	2022	Japan	2003–2019	285	Yes/No	HE	NA	NA	54.0%	69	18.6%
Fujino	2023	Japan	2008–2020	701	Bd2/Bd3	HE	NA	NA	NA	NA	NA
Kajiwara	2023	Japan	2009–2016	4673	Bd2/Bd3	NA	20× (objective lens)	0.785 mm^2^	59.3%	67.1	33.9%
Ebbehøj, M	2023	Denmark	2016–2019	765	Bd2/Bd3	NA	NA	NA	54.8%	68.5	29.1%

n, numbers; NA, not applicable.

### TB Scoring Methodology

Pooling studies found 5220 patients with high-grade TB and 19,736 patients with low-grade TB. For assessment, 39 studies used hematoxylin and eosin staining (HE), and 7 studies used immunohistochemical assessment with cytokeratin,^34^^,^^39^^,^^43^^,^^62^^,^^73^, ^74^, ^75^ the remaining 11 studies did not mention specific technologies.^10^^,^^28^^,^^52^^,^^53^^,^^63^^,^^67^^,^^69^^,^^78^, ^79^, ^80^, ^81^

The method of TB assessment was either defined by magnification (n = 35) or a specified area (n = 8), and seven of the studies included both specified area and magnification.^30^^,^^31^^,^^39^^,^^40^^,^^52^^,^^59^^,^^71^ The remaining 14 studies did not mention the assessment method. In line with international consensus, high-grade TB is defined as more than 10 TB per field of view. (Bd3, n = 11). In 34 studies, the high grade was defined as more than 5 TB (Bd2/Bd3). In the remaining 12 studies, the number of budding foci was not quantified, and TB was simply divided into two grades.

As shown in Tables A3 and A4, the quality scores of the 57 studies included in this meta-analysis are all over 60%, which proves that the overall quality of the literature included in this study is good. Among them, there are 47 high-quality studies. In particular, the scoring on assessment criteria 4 (assessment of biomarker expression), 5 (number of independent scorers noted), 8 (analysis of interactions), and 13 of 15 (reporting of estimated effect) is low. This is particularly noticeable in articles with a rating rate below 70%.

### The Relationship Between TB and LNM

**Figure 2:**
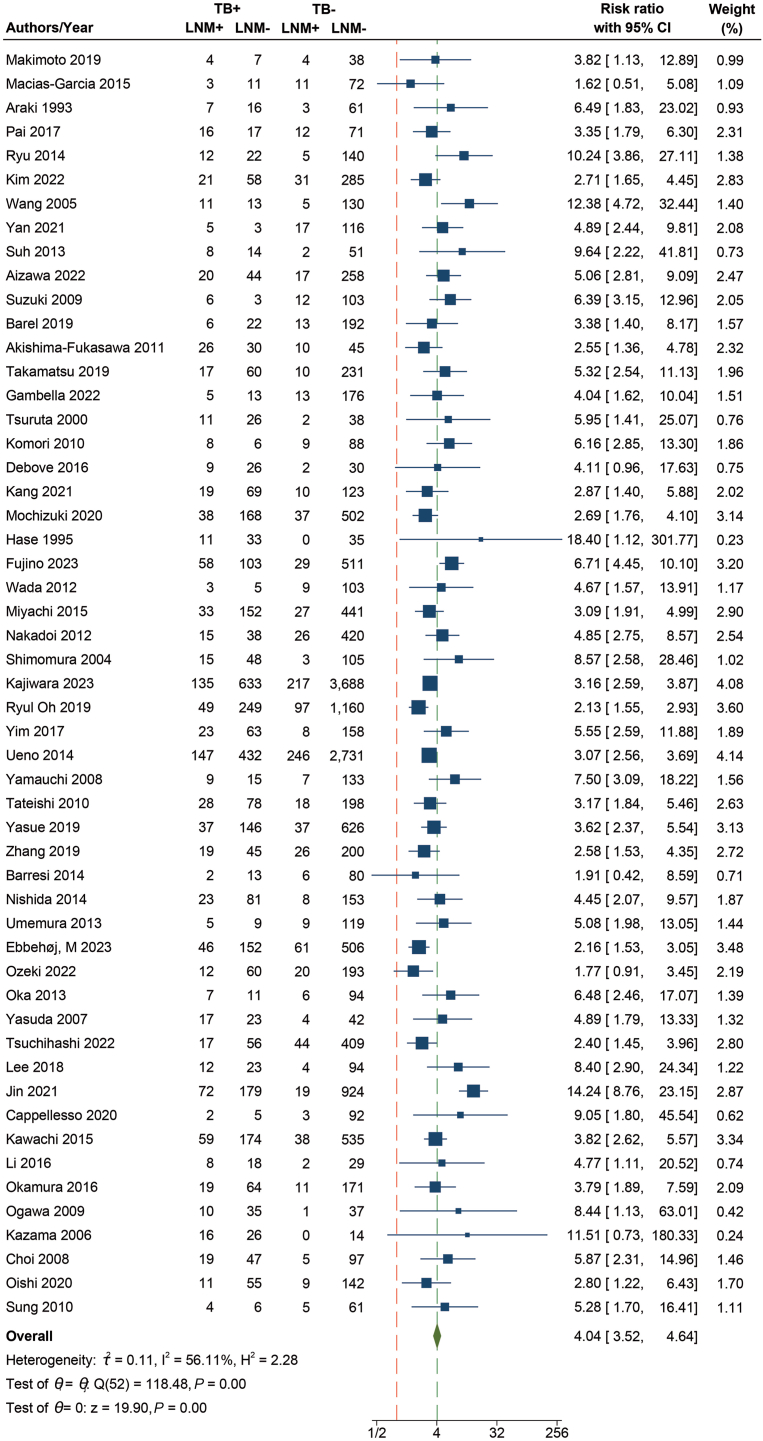
Forest plot with RR of risk of TB in relation to LNM.

A total of 53 studies were included, including 23,181 patients, of which 2425 patients had LNM. There was a significant association between TB and LNM (RR = 4.04 95% CI, 3.52–4.64, *P* < .001) (Figure 2), with significant heterogeneity using the random-effects model (I^2^ = 56.11%). Subgroup analyses (Table 2) were performed based on publication year, geographic location, number of budding foci, histological staining, tumor budding assessment (magnification and hotspot area, whether 0.785 mm^2^ was used), and tumor location to explore potential sources of heterogeneity. Compared to other studies, in studies where staining type or assessment was not described, significant heterogeneity was present (NA: I^2^ = 88.08%). In line, studies with a yes or no dichotomy for TB instead of Bd classification did show significant heterogeneity (NA: I^2^ = 74.86%). Studies that did not clarify the TB assessment method (Area and Magnification) also showed significant heterogeneity (NA: I^2^ = 79.12%) compared with other studies. Furthermore, we found significant differences in subgroup comparisons by year of publication and geographical location (*P* < .05).

**Table 2:** The Subgroup Analysis Between TB and LNM

Subgroups	RR (95% CI)	Heterogeneity (I^2^)	Studies	Patients	*P* Value between group
Geographic location					
Asia	4.22 (3.64–4.89)	59%	44	21,414	.01
Others	2.72 (2.08–3.57)	3.53%	9	1767	
Histological staining					
IHC	4.79 (3.36–6.83)	0%	7	1072	.46
HE	4.06 (3.54–4.67)	34.95%	39	13,223	
NA	3.31 (2.07–5.29)	88.08%	7	8886	
Number of budding foci					
Yes/No	5.51 (3.29–9.24)	74.86%	12	3210	.29
Bd2/Bd3	3.67 (3.22–4.17)	36.16%	31	17,378	
Bd3	4.17 (2.75–6.31)	51.93%	10	2593	
Tumor location					
Colorectal	4.02 (3.49–4.62)	57.24%	51	22,996	.42
Rectum	5.64 (2.52–12.62)	0%	2	185	
TB assessment (Area/Magnification)					
Area	3.53 (2.90–4.28)	16.66%	8	6822	.60
Magnification	3.82 (3.26–4.47)	31.66%	31	11,437	
NA	4.36 (2.97–6.40)	79.12%	14	4922	
Year					
<2015	4.96 (4.01–6.15)	32.28%	24	6929	.02
≥2015	3.58 (2.99–4.29)	65.42%	29	16,252	
TB assessment (0.785 mm^2^/No 0.785 mm^2^)					
0.785 mm^2^	3.68 (2.70–5.01)	37.45%	6	5900	.75
No 0.785 mm^2^	3.75 (3.25–4.32)	27.68%	33	12,359	
NA	4.36 (2.97–6.40)	79.12%	14	4922	

HE, hematoxylin-eosin staining; NA, not applicable; Local RE, local recurrence.

### The Relationship Between TB and Local Recurrence

**Figure 3:**
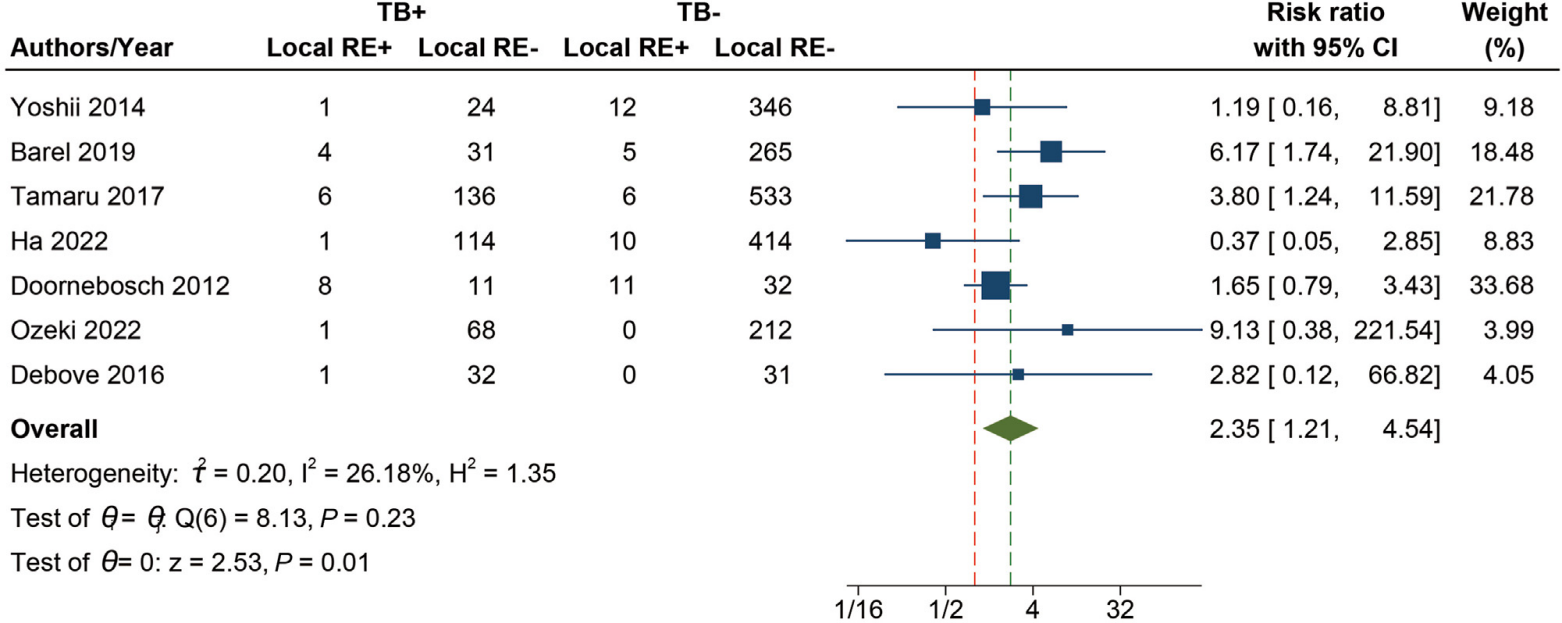
Forest plot with RR of risk of TB in relation to local RE. Local RE, local recurrence.

Seven studies were included, involving a total of 2360 patients, of whom 66 had local recurrence. There was a significant association between TB and local recurrence (RR = 2.35 95% CI, 1.21–4.54, *P* = .01) (Figure 3), no heterogeneity was observed (I^2^ = 26.18%). For completeness, we performed the same subgroup analysis as for the LNM but found no significant differences (Table A5; *P* > .05).

### Sensitivity Analysis

**Figure A1:**
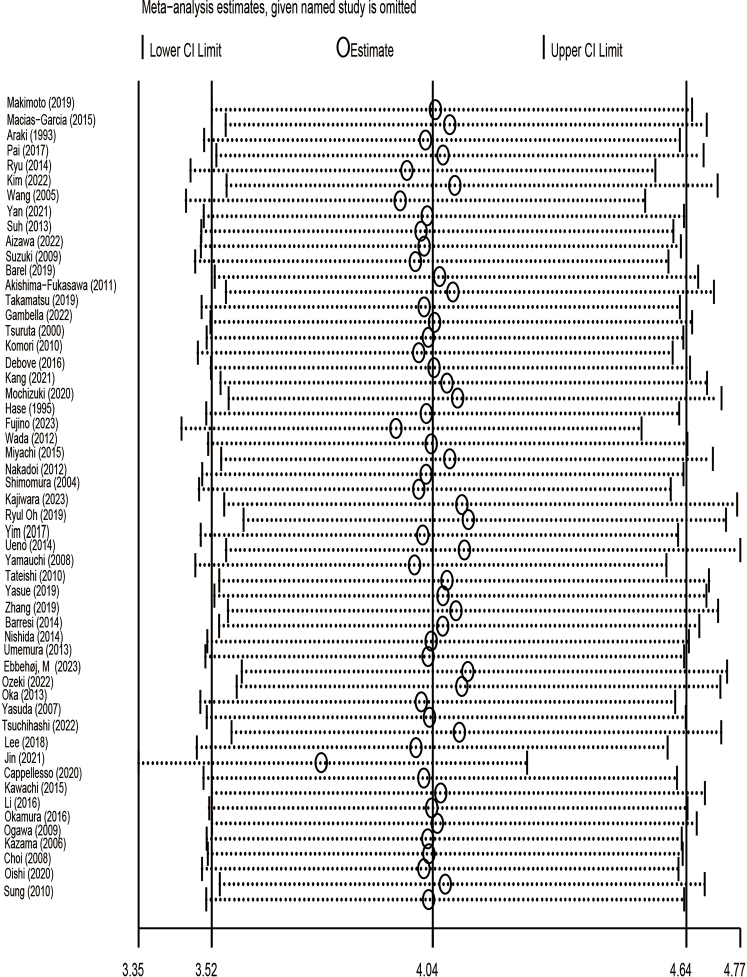
Sensitivity analysis between TB and LNM.

**Figure A2:**
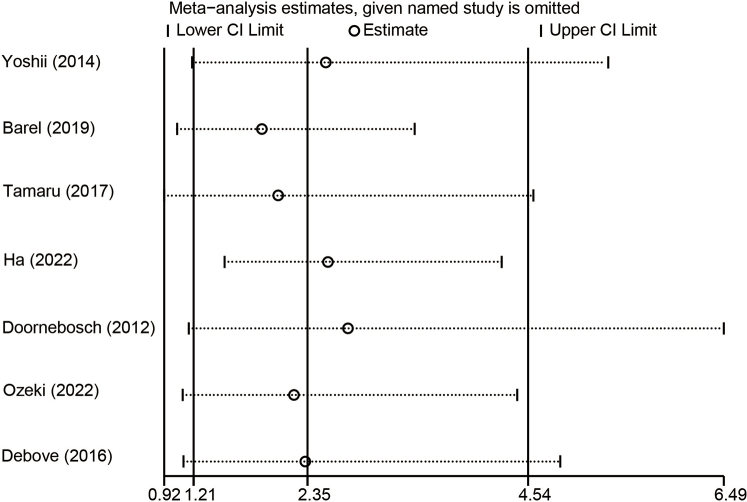
Sensitivity analysis between TB and local RE. Local RE, local recurrence.

Sensitivity analyses for LNM and local recurrence were performed to assess the stability of the results by excluding individual studies. These analyses showed that no individual studies influenced the value of the pooled analysis. The pooled results are stable and reliable (Figures A1 and A2). Additionally, sensitivity analyses for tumor site and magnification indicated that variations in these variables did not significantly affect the LNM results. However, for local recurrence, excluding studies based on tumor site or magnification led to changes in the findings. The observed RR with the remaining studies for rectum only tumors or magnification (200×) were lower and statistical significancy was not reached (Tables A6 and A7).

### Publication Bias

**Figure A3:**
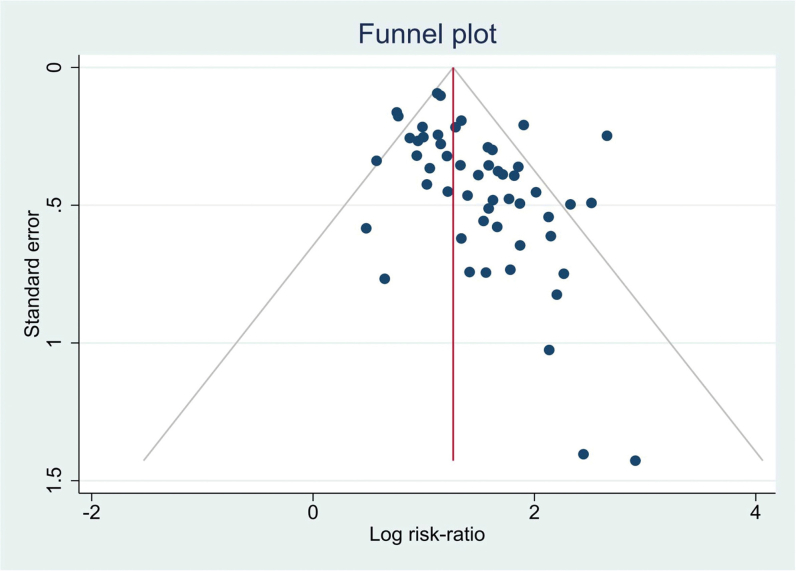
Funnel plot between tumor TB and LNM.

**Figure A4:**
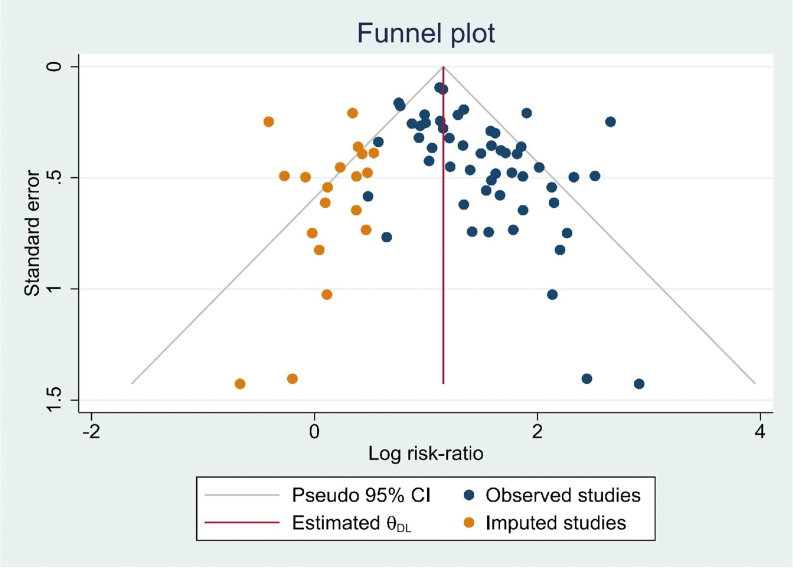
Funnel plot obtained with the trim and fill method for the analysis of TB and LNM.

**Figure A5:**
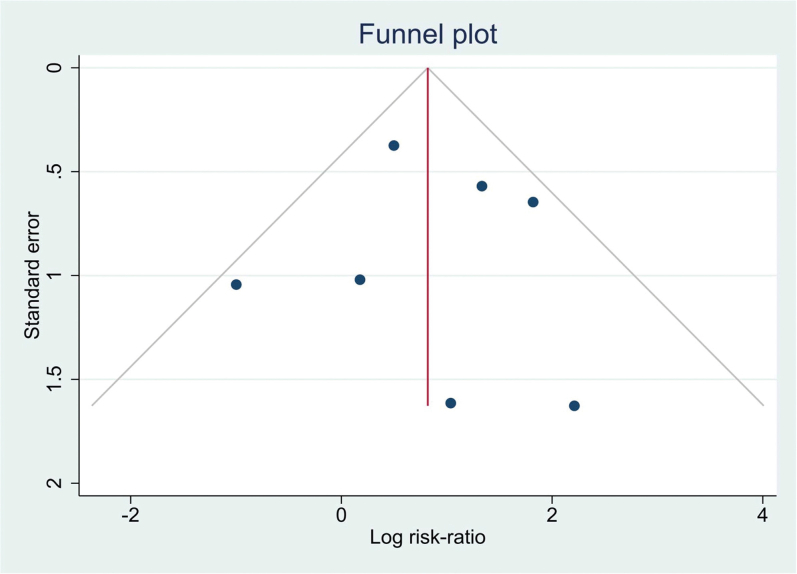
Funnel plot between TB and local RE. Local RE, local recurrence.

We created separate funnel plots for these 2 results to test for possible publication bias in the study (Figures A3 and A5). Visual inspection suggests potential publication bias for LNM, but not local recurrence. The results were verified by Egger's test (Egger value of LNM and Local recurrence: *P* = .001 and *P* = .9637). Nevertheless, reanalysis of RR value adjusted by the number of articles by the trimming and filling method showed still a significantly increased RR (RR = 3.181, 95% CI, 2.735–3.699, *P* < .001), indicating this potential publication bias did not influence the results (Figure A4)

## Discussion

In this meta-analysis, we investigated the relationship between TB and LNM as well as local recurrence in pT1 CRC. A total of 57 retrospective studies were included. The presence of TB was significantly predictive for both LNM (RR = 4.04, 95% CI, 3.52–4.64, *P* < .001), and local recurrence (RR = 2.35 95% CI, 1.21–4.54, *P* = .01). The aggressiveness of tumors with high levels of TB is supported by biological evidence. TB is a dynamic process of tumor cells and tumor mass separating from one another and is closely related to epithelial–mesenchymal transition (EMT). Some characteristics of EMT, such as cell migration and invasion, cytoskeletal rearrangement, reduced expression of key EMT regulators E-cadherin and membrane β-catenin, and Laminin 5ɣ2 positivity, are associated with TB^82^ and have been linked to many adverse features, recurrence, and poor overall survival rates.^83^

The current study analyzed over 20,000 patients and found that high-grade TB increases the risk of LNM. A total of 2425 patients in the study experienced LNM, resulting in an overall incidence of 10.5%, consistent with previous reports.^11^ This focused meta-analysis confirmed earlier studies and highlighted the importance of a standardized approach, as advocated by the International Tumor Budding Consensus Conference (ITBCC),^13^ as has been established in a consensus study with a panel of gastrointestinal pathology experts.^84^

This study also investigated the relationship between TB and local recurrence in pT1 CRC, finding that high-grade TB increases the risk of local recurrence, with an RR value of 2.3, which is in line with a 2016 meta-analysis, which included 34 studies on colorectal cancer of different stages (including 12 studies on recurrence), also found a significant correlation between high-grade TB and disease recurrence, with an OR of 5.50.^85^ However, this study included all stages.

In the meta-analysis for LNM, heterogeneity was observed (I^2^ = 56.11%), which could be explained by the studies that provided limited methodological information. Additionally, the combined results of 2 subgroup analyses showed significant differences in geographical location (*P* = .01) and publication year (*P* = .02), with higher RR values in studies from Asia (RR = 4.22). The studies included in this analysis were mainly from Asia, Europe, and North America. Most of the Asian literature came from Japan, which accounted for 68% of the Asian studies. In the 2010 Japanese Society for Cancer of the Colon and Rectum guidelines, TB was identified as a risk factor for LNM and was incorporated into the Japanese CRC treatment guidelines.^86^

This has made routine TB detection and reporting in Japan possible. In contrast, a study primarily involving pathology reports from European and North American countries showed that CRC pathology reporting guidelines vary, with 28.7% of participants having never reported TB in pathology reports.^87^ This suggests that the frequency of TB detection and reporting may be lower in Western countries. Differences in the reporting of TB detection (routine vs experts) may be a key factor contributing to the regional variations observed in subgroup analysis outcomes.

There were also significant differences in publication years, with a lower RR value for studies published after 2015 (RR = 3.58). This can be attributed to the ITBCC, which standardized the definition and diagnostic criteria and recommended its inclusion in CRC guidelines, protocols, and staging systems.^13^ The standardization of TB diagnosis and treatment has also facilitated the development of numerous TB-related cancer guidelines, enabling patients to receive adequate treatment at an early stage.^20^^,^^21^

According to the ITBCC, for most of the studies the evaluation of bud counts was based on hematoxylin and eosin staining. However, severe inflammation may mask the presence of TB, IHC might be considered.^39^^,^^82^^,^^88^ Of the 57 studies included in this review, only 7 studies used IHC (cytokeratin). Cytokeratin can increase the bud count accuracy and repeatability.^39^^,^^89^ Accumulating evidence has also shown that IHC does improve interobserver consistency and reproducibility compared to HE staining.^90^^,^^91^

At the same time, there are also contrary opinions. Two studies have shown that if you encounter pseudo-tumor budding caused by mechanical damage or inflammation and other related reasons, IHC may not be able to completely distinguish it from real TB, and the evaluation effect is not as good as HE staining.^73^^,^^92^ Therefore, the selection of the appropriate histochemical assessment method is still controversial.

A meta-analysis is limited by the quality and availability of underlying studies. We observe variation in methodology and cut-off values, while global guidelines are available. Despite this, we were able to collect a large patient cohort, thereby reducing the likelihood of false-negative results (type II errors). Sensitivity analysis demonstrated the stability of our pooled results. Compared to previous studies on TB and LNM, our study included a larger number of patients and optimized the inclusion criteria, further validating the relationship between TB and LNM and improving the accuracy of the results. For local recurrence, a limited number of studies (n = 7) were available. This may be a key reason for the instability of the results after conducting sensitivity analyses based on tumor site and magnification. With increased numbers of local excisions, due to implementation of population screening programs and improved endoscopic modalities, it is likely that more data will become available in the near future.

## Conclusion

This meta-analysis demonstrates that TB is a strong predictor of both LNM and local recurrence in pT1 CRC. This meta-analysis provides additional information on the need for additional surgery after local resection of pT1 CRC. Therefore, TB should be routinely included in the evaluation of CRC, which will improve patient treatment decisions.
