# Supplementary material for: Tumor Budding as a Risk Factor for Lymph Node Metastasis and Local Recurrence in pT1 Colorectal Cancer: A Systematic Review and Meta-Analysis
Source: Gastro Hep Adv. 2025 May 27;4(9):100713. doi: 10.1016/j.gastha.2025.100713 (PMC12320163; doi:10.1016/j.gastha.2025.100713)

**Supplementary material**

**Supplementary** **Table 1:** Search strategies.

**Ovid MEDLINE(R) ALL <1946 to May 24, 2023>**

| 1 | exp Colorectal Neoplasms/ | 235870 |
| --- | --- | --- |
| 2 | ((colorect* or colo-rect* or colon* or sigmoid or rectal or rectum) adj3 (neoplasm* or cancer* or carcinoma* or adenocarcinoma* or adeno-carcinoma* or tumor* or tumour* or malignan*)).mp. | 316914 |
| 3 | ((malignan* or adenoma*) adj3 (colorect* or colo-rect* or colon* or sigmoid or rectal or rectum) adj3 polyp*).mp. | 2123 |
| 4 | ((colorect* or colo-rect* or colon* or sigmoid or rectal or rectum) adj3 polyp*).mp. | 19465 |
| 5 | ((submucosa* or sub-mucosa*) adj2 (invasion or depth)).mp. | 1404 |
| 6 | or/1-5 | 332024 |
| 7 | (budding or sprouting).mp. | 34098 |
| 8 | 6 and 7 | 966 |

**Embase <1974 to 2023 May 24>**

| 1 | exp colorectal tumor/ | 455364 |
| --- | --- | --- |
| 2 | ((colorect* or colo-rect* or colon* or sigmoid or rectal or rectum) adj3 (neoplasm* or cancer* or carcinoma* or adenocarcinoma* or adeno-carcinoma* or tumor* or tumour* or malignan*)).mp. | 500154 |
| 3 | ((malignan* or adenoma*) adj3 (colorect* or colo-rect* or colon* or sigmoid or rectal or rectum) adj3 polyp*).mp. | 3379 |
| 4 | ((colorect* or colo-rect* or colon* or sigmoid or rectal or rectum) adj3 polyp*).mp. | 38634 |
| 5 | ((submucosa* or sub-mucosa*) adj2 (invasion or depth)).mp. | 2797 |
| 6 | or/1-5 | 526231 |
| 7 | (budding or sprouting).mp. | 45184 |
| 8 | 6 and 7 | 1842 |
| 9 | limit 8 to conference abstract | 683 |
| 10 | 8 not 9 | 1159 |

**Supplementary** **Table 2:** Selection of quality assessment method.

**LNM (Lymph Node Metastasis):**

| 1. Describe patient characteristics, and inclusion and exclusion criteria. |
| --- |
| 2. Describe treatment details. |
| 3. Describe the type of material used. |
| 4. Specify how expression of the biomarker was assessed. |
| 5. Describe the number of independent (blinded) scorers and how they scored. |
| 6. State the method of case selection, study design, origin of the cases, and time frame. |
| 7. Specify all applied statistical methods. |
| 8. Describe how interactions with other clinical/pathological factors were analyzed. |
| 9. Describe the number of patients included in the analysis and reason for dropout. |
| 10. Report patient/disease characteristics (including the biomarker of interest) with the number of missing values. |
| 11. Describe the interaction of the biomarker of interest with established prognostic variables. |
| 12. Include at least 90 % of initial cases included in univariate and multivariate analyses. |
| 13. Report the estimated effect (relative risk/odds ratio, confidence interval, and p value) in univariate analysis. |

**Local RE (Tumor Local recurrence)**

| 1. Describe patient characteristics, and inclusion and exclusion criteria. |
| --- |
| 2. Describe treatment details. |
| 3. Describe the type of material used. |
| 4. Specify how expression of the biomarker was assessed. |
| 5. Describe the number of independent (blinded) scorers and how they scored. |
| 6. State the method of case selection, study design, origin of the cases, and time frame. |
| 7.Describe the end of the follow-up period and median follow-up time. |
| 8.Define all clinical endpoints examined. |
| 9. Specify all applied statistical methods. |
| 10. Describe how interactions with other clinical/pathological factors were analyzed. |
| 11. Describe the number of patients included in the analysis and reason for dropout. |
| 12. Report patient/disease characteristics (including the biomarker of interest) with the number of missing values. |
| 13. Describe the interaction of the biomarker of interest with established prognostic variables. |
| 14. Include at least 90 % of initial cases included in univariate and multivariate analyses. |
| 15. Report the estimated effect (relative risk/odds ratio, confidence interval, and p value) in univariate analysis. |

**Supplementary** **Table 3:** Quality assessment results of the relationship between TB and LNM.

| **Author(year)** | **1** | **2** | **3** | **4** | **5** | **6** | **7** | **8** | **9** | **10** | **11** | **12** | **13** |  | **Score** | **Score ration** |
| --- | --- | --- | --- | --- | --- | --- | --- | --- | --- | --- | --- | --- | --- | --- | --- | --- |
| Makimoto 2019 | Good | Good | Good | Poor | Poor | Good | Good | Good | Good | Good | Good | Good | Poor |  | 11.5 | 88% |
| Macias-Garcia 2015 | Good | Good | Poor | Poor | No | Good | Good | No | Good | Good | Good | Good | Poor |  | 9.5 | 73% |
| Araki 1993 | Good | Good | Good | Poor | No | Good | Good | No | Good | Good | Good | Good | No |  | 9.5 | 73% |
| Pai 2016 | Good | Good | Good | Good | No | Good | Good | Good | Good | Good | Good | Good | Poor |  | 11.5 | 88% |
| Ryu 2014 | Good | Good | Good | Good | Good | Good | Good | Good | Poor | Good | Good | Good | Good |  | 12.5 | 96% |
| Kim 2022 | Good | Good | Good | Good | Good | Good | Good | Good | Poor | Good | Good | Good | Poor |  | 12 | 92% |
| Wang 2005 | Good | Good | Good | Poor | Poor | Good | Good | Good | Good | Good | Good | Good | Poor |  | 11.5 | 88% |
| Yan 2021 | Good | Good | Good | Poor | Good | Good | Good | Good | Good | Good | Good | Good | Poor |  | 12 | 92% |
| Suh 2013 | Good | Good | Good | Good | Good | Good | Good | Good | Good | Good | Good | No | Poor |  | 11.5 | 88% |
| Aizawa 2022 | Good | Good | Good | Poor | Good | Good | Good | Good | Good | Good | Good | Good | Good |  | 12.5 | 96% |
| Suzuki 2009 | Good | Good | Good | Good | Poor | Good | Good | Good | Good | Good | Good | Good | Poor |  | 12 | 92% |
| Barel 2019 | Good | Poor | Good | Good | Good | Good | Good | No | Good | Good | Good | No | Poor |  | 10 | 77% |
| Akishima-Fukasawa 2011 | Good | Good | Good | Good | Good | Good | Good | Good | Poor | Poor | Good | Good | Poor |  | 11.5 | 88% |
| Takamatsu 2018 | Good | Good | Good | Good | Good | Good | Good | Good | Poor | Good | Good | No | Poor |  | 11 | 85% |
| Gambella 2022 | Good | Good | Good | Good | Good | Good | Good | No | Good | Good | Good | Good | Good |  | 12 | 92% |
| Tsuruta 2000 | Poor | Good | Good | Poor | No | Good | Good | No | Good | Good | Good | Good | Poor |  | 9.5 | 73% |
| Komori 2010 | Poor | Good | Good | Good | Poor | Good | Good | Good | Good | Good | Good | Good | No |  | 11 | 85% |
| Debove 2016 | Good | Good | Poor | Good | Poor | Good | Good | Good | Good | Good | Good | No | Poor |  | 10.5 | 81% |
| Kang 2021 | Good | Good | Good | Good | Poor | Good | Good | No | Good | Good | Good | Good | Good |  | 11.5 | 88% |
| Mochizuki 2020 | Good | Good | Good | Good | No | Good | Good | Good | Good | Good | Good | No | Poor |  | 10.5 | 81% |
| Hase 1995 | Good | Good | Good | Poor | Poor | Good | Good | No | Good | Good | Good | Good | Poor |  | 10.5 | 81% |
| Fujino 2023 | Good | Good | Good | Poor | No | Good | Good | Good | Good | Good | Good | No | Good |  | 10.5 | 81% |
| Wada 2012 | Good | Good | Good | Good | No | Good | Good | Good | Good | Good | Good | Good | Poor |  | 11.5 | 88% |
| Miyachi 2016 | Good | Good | Good | Good | Poor | Good | Good | Good | Good | Good | Good | Good | Good |  | 12.5 | 96% |
| Nakadoi 2011 | Good | Good | Good | Good | Poor | Good | Good | Good | Good | Good | Good | Good | Poor |  | 12 | 92% |
| Shimomura2004 | Good | Good | Good | Poor | Poor | Good | Good | No | Good | Good | Good | Good | Poor |  | 10.5 | 81% |
| Kajiwara 2023 | Good | Good | Poor | Poor | No | Good | Good | Good | Good | Good | Good | Good | Poor |  | 10.5 | 81% |
| Ryul Oh2019 | Good | Good | Poor | Poor | No | Good | Good | Good | Good | Good | Good | Good | Good |  | 11 | 85% |
| Yim 2017 | Poor | Good | Good | Good | Good | Good | Good | No | Good | Good | Good | Good | Poor |  | 11 | 85% |
| Ueno 2013 | Poor | Good | Good | Good | Poor | Good | Good | Good | Good | Good | Good | Good | Poor |  | 11.5 | 88% |
| Yamauchi 2008 | Poor | Good | Poor | Good | Good | Good | Good | Good | Good | Good | Good | Good | Good |  | 12 | 92% |
| Tateishi 2010 | Good | Good | Good | Poor | Good | Good | Good | Good | Good | Good | Good | Good | Poor |  | 12 | 92% |
| Yasue 2019 | Good | Good | Good | Good | Poor | Good | Good | Good | Good | Good | Good | Good | Poor |  | 12 | 92% |
| Zhang 2019 | Poor | Good | Good | Good | Good | Good | Good | Good | Good | Good | Good | Good | Good |  | 12.5 | 96% |
| Barresi 2014 | Poor | Good | Good | Good | No | Good | Good | No | Good | Good | Good | Good | Good |  | 10.5 | 81% |
| Nishida 2014 | Poor | Good | Good | Good | Good | Good | Good | Good | Good | Good | Good | Good | Good |  | 12.5 | 96% |
| Umemura 2013 | Good | Good | Good | Good | Good | Good | Good | Good | Good | Good | Good | Good | Poor |  | 12.5 | 96% |
| Ebbehøj, M 2023 | Good | Good | Good | Poor | No | Good | Good | Good | Good | Good | Good | No | Good |  | 10.5 | 81% |
| Ozeki 2022 | Good | Good | Good | Poor | Poor | Good | Good | Good | Good | Good | Good | No | Poor |  | 10.5 | 81% |
| Oka 2013 | Good | Good | Good | Good | No | Good | Good | Good | Good | Good | Good | Good | Poor |  | 11.5 | 88% |
| Yasuda 2007 | Good | Good | Good | Poor | No | Good | Good | Good | Good | Good | Good | Good | Poor |  | 11 | 85% |
| Tsuchihashi 2022 | Good | Good | Good | No | Good | Good | Good | Good | Good | Good | Good | Good | Good |  | 12 | 92% |
| Lee 2018 | Good | Good | Good | Good | Good | Good | Good | Good | Good | Good | Good | Good | Poor |  | 12.5 | 96% |
| Jin 2021 | Good | Good | Good | No | No | Good | Good | No | Good | Good | Good | Good | Poor |  | 9.5 | 73% |
| Cappellesso 2020 | Poor | Good | Good | Good | No | Good | Good | Good | Good | Good | Good | Good | Poor |  | 11 | 85% |
| Kawachi 2015 | Poor | Good | Good | Good | Good | Good | Good | Good | Good | Good | Good | Good | Poor |  | 12 | 92% |
| Li 2016 | Good | Good | Poor | Poor | Good | Good | Good | No | Good | Good | Good | No | Poor |  | 9.5 | 73% |
| Okamura 2016 | Good | Good | Good | Good | Good | Good | Good | Good | Good | Good | Good | Good | Poor |  | 12.5 | 96% |
| Ogawa 2009 | Poor | Good | Good | Good | Good | Good | Good | Good | Good | Good | Good | No | Poor |  | 11 | 85% |
| Kazama 2006 | Poor | Good | Good | Poor | Good | Good | Good | No | Good | Good | Good | Good | Poor |  | 10.5 | 81% |
| Choi 2008 | Good | Good | Good | Poor | Good | Good | Good | Good | Good | Good | Good | Good | Poor |  | 12 | 92% |
| Oishi 2020 | Good | Good | Good | Poor | No | Good | Good | Good | No | Good | Good | Good | Good |  | 10.5 | 81% |
| Sung 2010 | Poor | Good | Poor | Poor | No | Good | Good | Good | Good | Good | Good | Good | Poor |  | 10 | 77% |

Low-quality documents: ≤ 60%; Medium-quality documents: 60%-80%; High-quality documents:>80%.

**Supplementary** **Table 4:** Quality assessment results of the relationship between TB and Local RE.

| **Local RE** | **1** | **2** | **3** | **4** | **5** | **6** | **7** | **8** | **9** | **10** | **11** | **12** | **13** | **14** | **15** |  | **Score** | **Score ration** |
| --- | --- | --- | --- | --- | --- | --- | --- | --- | --- | --- | --- | --- | --- | --- | --- | --- | --- | --- |
| Yoshii 2013 | Good | Good | Poor | Poor | Good | Good | Good | Good | Good | No | Good | Good | Good | Good | Poor |  | 12.5 | 83% |
| Barel 2019 | Good | Poor | Good | Good | Good | Good | No | Poor | Good | No | Good | Good | Good | No | Poor |  | 10.5 | 70% |
| Tamaru 2016 | Good | Good | Poor | Good | Good | Good | Good | Good | Good | No | Good | Good | Good | No | No |  | 11.5 | 77% |
| Ha 2022 | Good | Good | Poor | Poor | Poor | Good | Good | Good | Good | Good | Good | Good | Good | Good | Good |  | 13.5 | 90% |
| Doornebosch 2012 | Poor | Good | Poor | Poor | Good | Good | Good | Poor | Good | No | Good | Good | Good | Good | Poor |  | 11.5 | 77% |
| Ozeki 2022 | Good | Good | Good | Poor | Poor | Good | No | Good | Good | Good | Good | Good | Good | No | Poor |  | 11.5 | 77% |
| Debove 2016 | Good | Good | Poor | Good | Poor | Good | Good | Good | Good | Good | Good | Good | Good | No | Poor |  | 12.5 | 83% |

Low-quality documents: ≤ 60%; Medium-quality documents: 60%-80%; High-quality documents:>80%.

**Supplementary** **Table 5:** Subgroup analysis of TB and Local RE.

| **Subgroups** | | **RR （95%,CI)** | **Heterogeneity (I2)** | **Number of studies** | **Number of patients** | ***P* value between group** |
| --- | --- | --- | --- | --- | --- | --- |
| Area | Asia | 1.88 (0.55-6.40) | 39.65% | 4 | 1922 | 0.65 |
|  | Others | 2.71 (1.04-7.05) | 36.46% | 3 | 438 |  |
| Histological staining | IHC | 2.82 (0.12-66.82) | 100% | 1 | 67 | 0.17 |
|  | HE | 6.51 (2.01-21.13) | 0% | 2 | 594 |  |
|  | NA | 1.70 (0.80-3.62) | 29.26% | 4 | 1699 |  |
| Assessment of budding (Cut-off value) | Yes/No | 9.13 (0.38-4.30) | 100.00% | 1 | 285 | 0.30 |
|  | Bd2/Bd3 | 2.63 (1.33-5.20) | 29.60% | 4 | 1461 |  |
|  | Bd3 | 0.70 (0.11-4.50) | 10.84% | 2 | 614 |  |
| Tumor location | Colorectal | 2.65 (0.98-7.12) | 40.62% | 5 | 2231 | 0.47 |
|  | Rectum | 1.69 (0.83-3.46) | 0% | 2 | 129 |  |
| Budding assessment (Area/Magnification) | Area | 6.17 (1.74-21.90) | 100.00% | 1 | 309 | 0.16 |
|  | Magnification | 1.79 (0.97-3.29) | 7.45% | 5 | 1766 |  |
|  | NA | 9.13 (0.38-221.54) | 100% | 1 | 285 |  |
| Year | ＜2015 | 1.58 (0.80-3.15) | 0% | 2 | 451 | 0.26 |
|  | ≥2015 | 3.13 (1.18-8.26) | 31.06% | 5 | 1909 |  |
| Budding assessment (0.785mm2/No 0.785mm2) | 0.785mm2 | 6.17 (1.74-21.90) | 100% | 1 | 309 | 0.16 |
|  | No 0.785mm2 | 1.79 (0.97-3.29) | 7.45% | 5 | 1766 |  |
|  | NA | 9.13 (0.38-221.54) | 100% | 1 | 285 |  |

NA: not applicable; HE: hematoxylin-eosin staining; IHC: immunohistochemistry; mm2: square millimeters; Local RE: local recurrence; LNM: lymph node metastasis.

**Supplementary** **Table 6:** Sensitivity analysis for tumor site and magnification in LNM.

| **Groups** | | **RR (95%CI)** | **Heterogeneity (I2)** | **Studies** | **Patients** | ***P* value** |
| --- | --- | --- | --- | --- | --- | --- |
| Tumor site | No Rectum | 4.02 (3.49-4.62) | 57.24% | 51 | 22996 | 0 |
|  | No Colorectum | 5.64 (2.52-12.62) | 0% | 2 | 185 | 0 |
| Magnification | No 50x | 4.02 (3.50-4.61) | 57.06% | 51 | 23046 | 0 |
|  | No 100x | 4.03 (3.51-4.63) | 56.78% | 52 | 23010 | 0 |
|  | No 200x | 4.02 (3.34-4.84) | 63.30% | 31 | 17242 | 0 |
|  | No 250x | 4.08 (3.56-4,69) | 56.28% | 52 | 23084 | 0 |
|  | No 400x | 3.98 (3.46-4.58) | 57.37% | 50 | 22740 | 0 |
|  | No NA | 4.23 (3.49-5.14) | 38.67% | 29 | 16398 | 0 |

NA: not applicable; LNM: lymph node metastasis.

**Supplementary** **Table 7:** Sensitivity analysis for tumor site and magnification in Local RE.

| **Groups** | | **RR (95%CI)** | **Heterogeneity (I2)** | **Studies** | **Patients** | ***P* value** |
| --- | --- | --- | --- | --- | --- | --- |
| Tumor site | No Rectum | 2.65 (0.98-7.12) | 40.62% | 5 | 2231 | 0.05 |
|  | No Colorectum | 1.69 (0.83-3.46) | 0% | 2 | 129 | 0.15 |
| Magnification | No 200x | 2.83 (1.24-6.50) | 21.75% | 4 | 723 | 0.01 |
|  | No NA | 1.47 (0.36-5.95) | 51.76% | 3 | 1637 | 0.59 |

NA: not applicable; Local RE: local recurrence.

**Supplementary** **Figure 1:** Sensitivity analysis between TB and LNM.


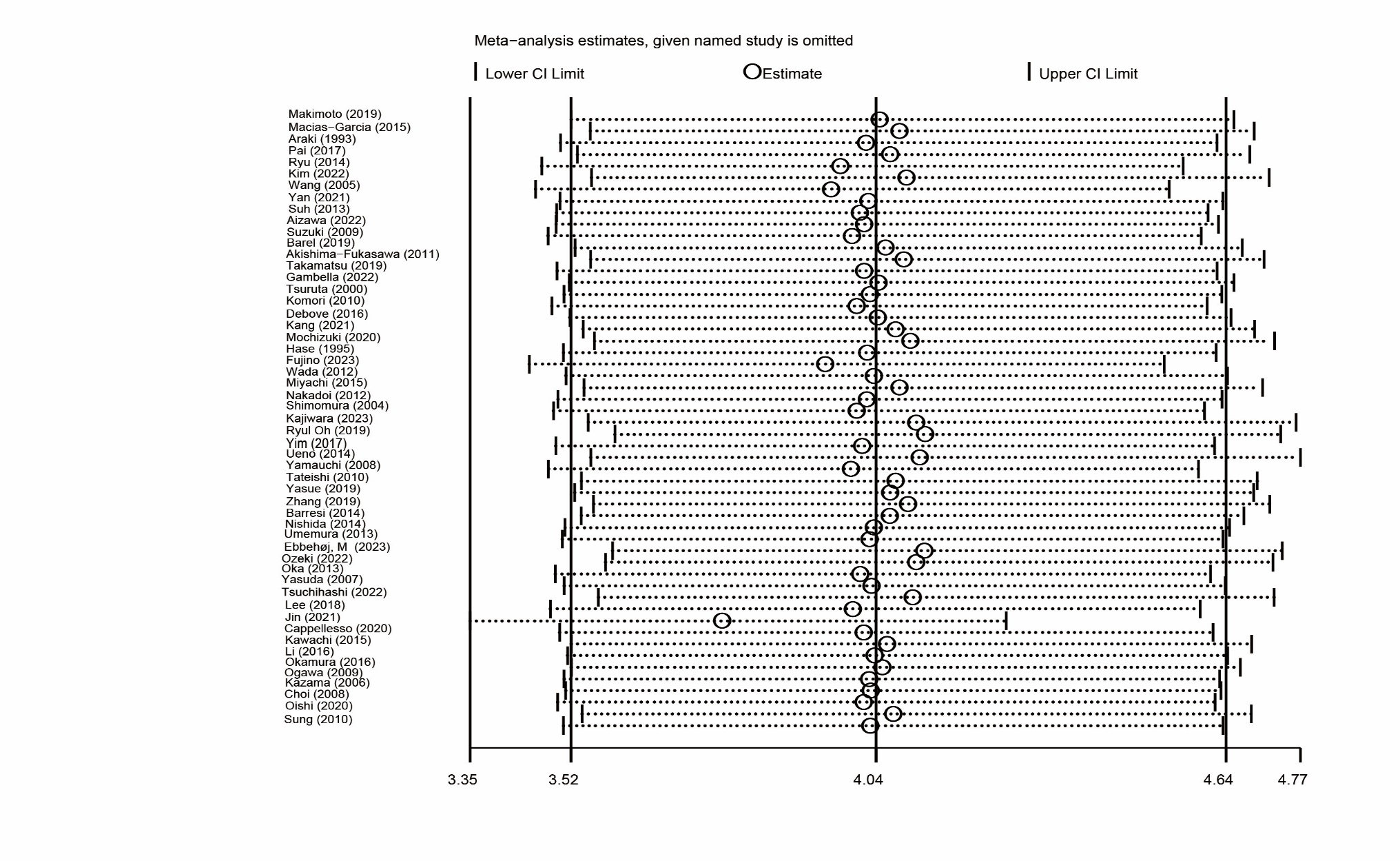


**Supplementary** **Figure 2:** Sensitivity analysis between TB and Local RE.


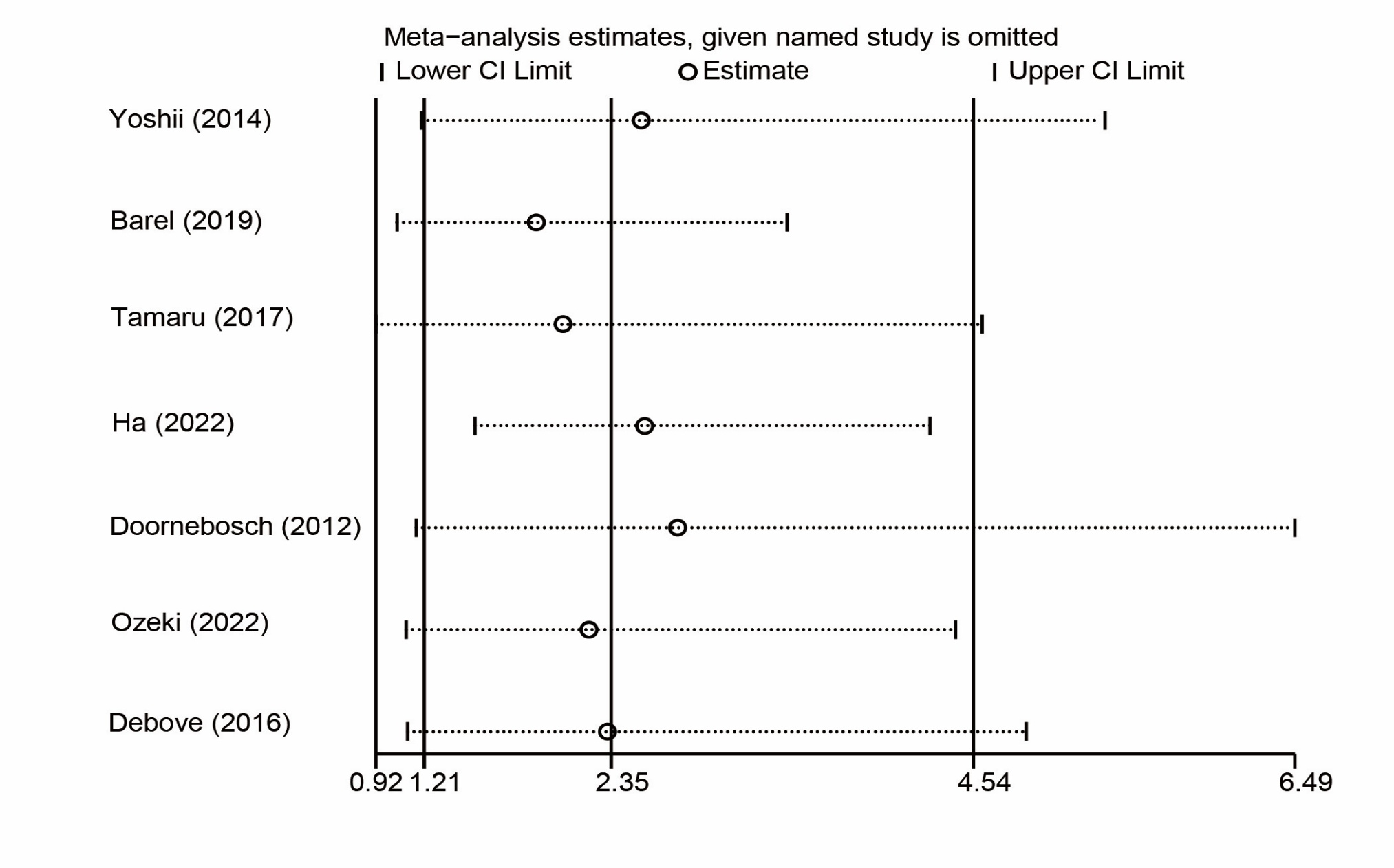


**Supplementary** **Figure 3:** Funnel plot between tumor TB and LNM.


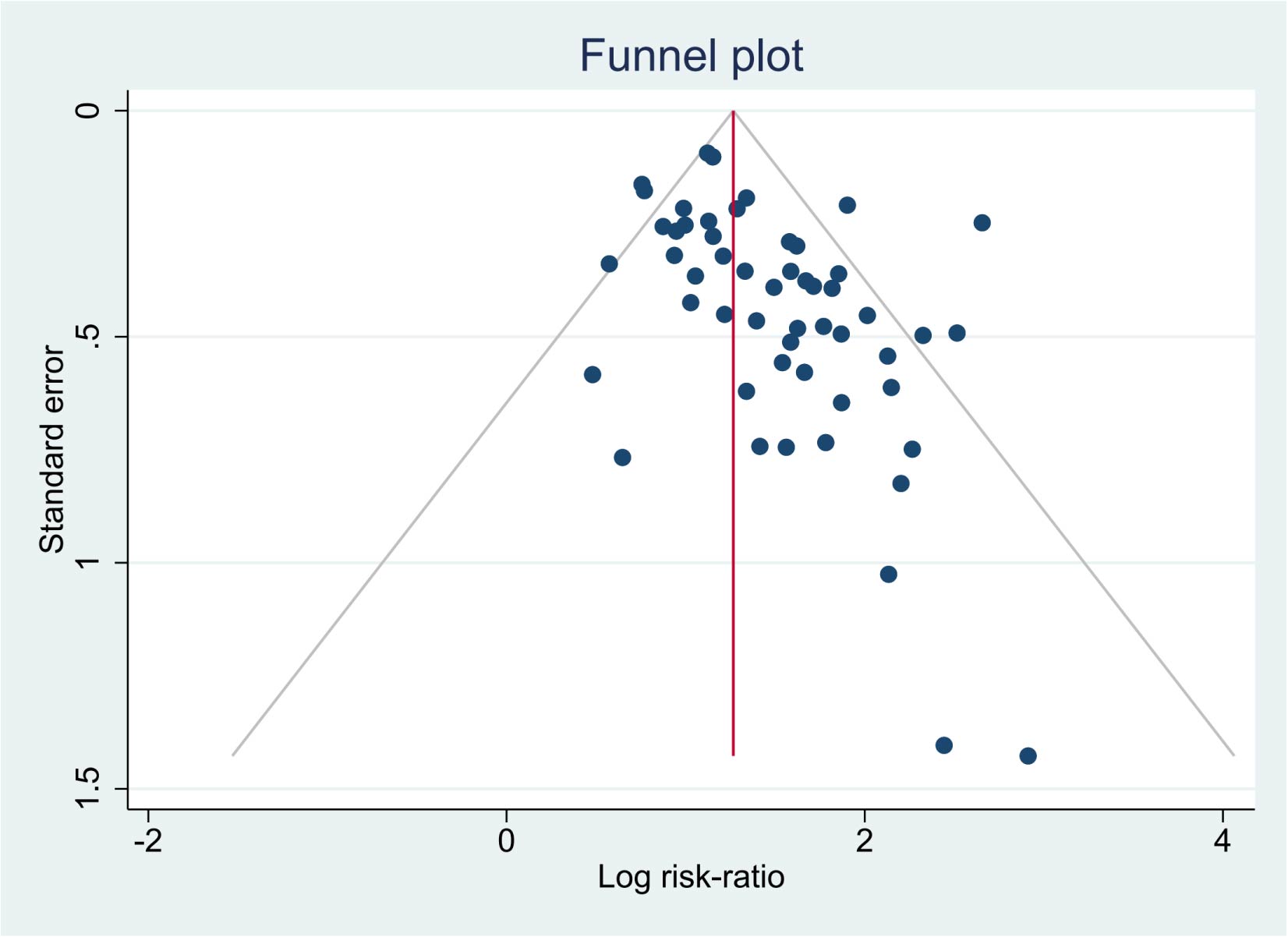


**Supplementary** **Figure 4:** Funnel plot obtained with the trim and fill method for the analysis of TB and LNM.


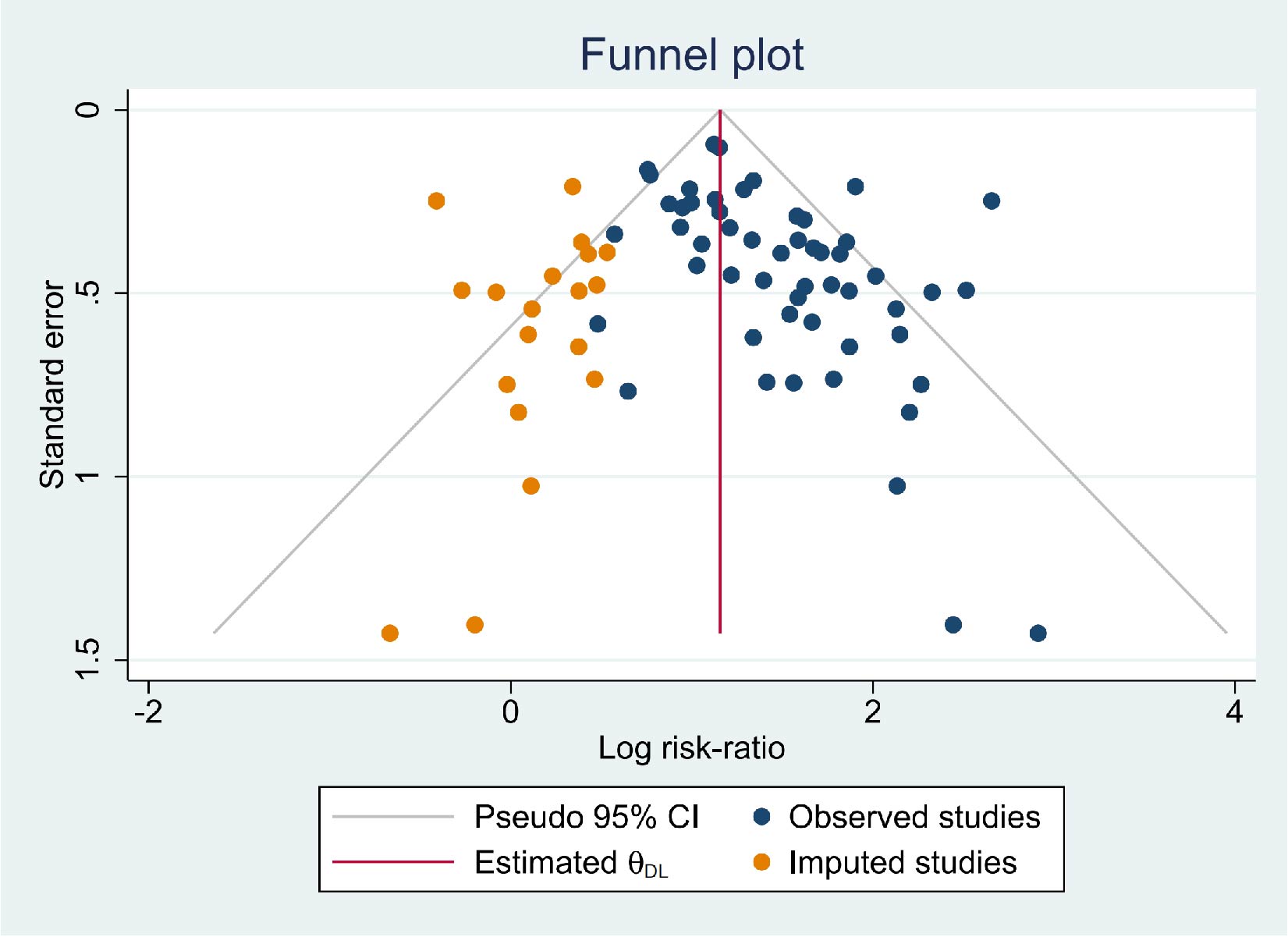


**Supplementary** **Figure 5:** Funnel plot between TB and Local RE.


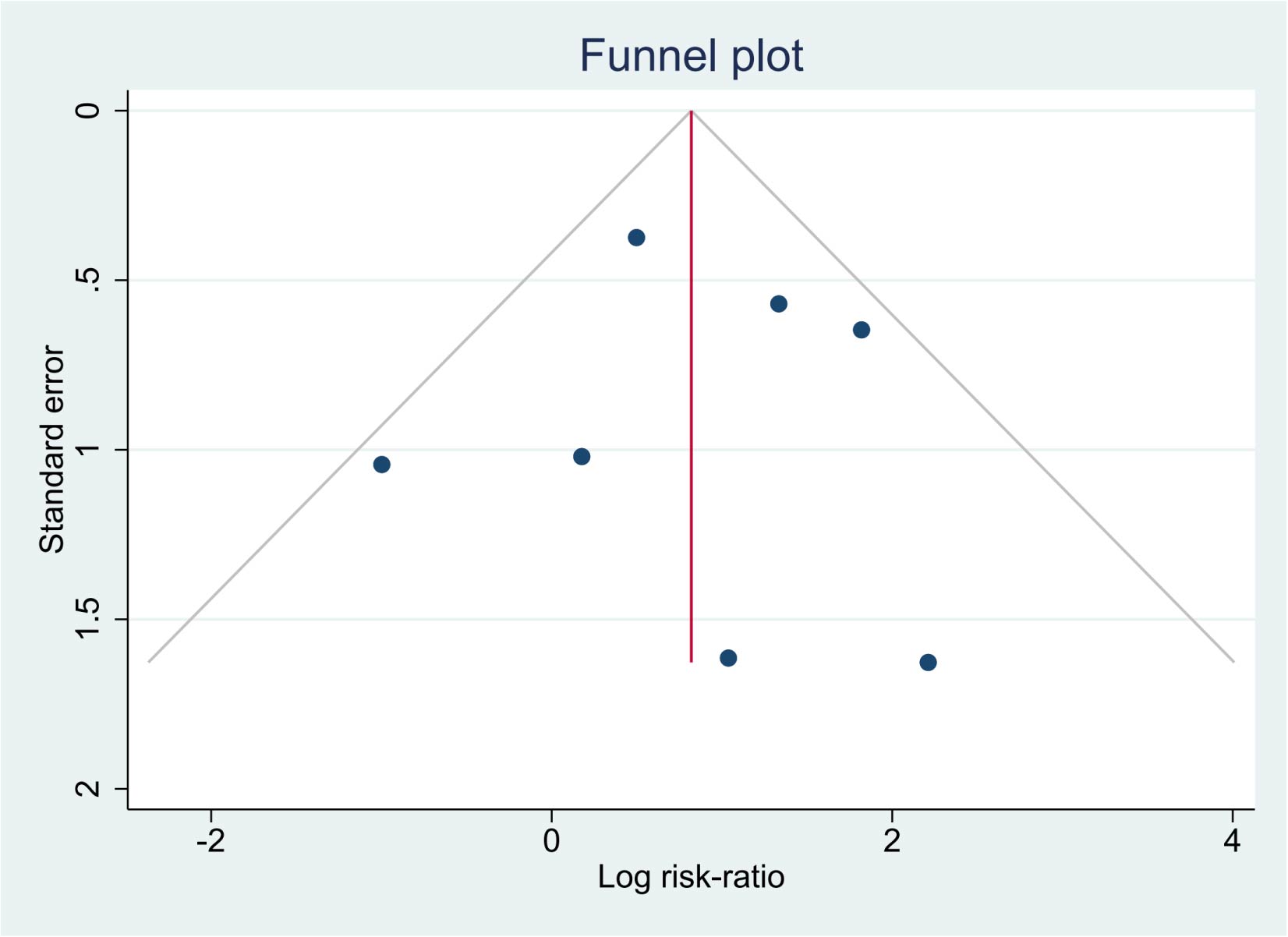

Supplement: Supplementary Material [file mmc1.docx]
